# Account of the Discovery, by Purkinje and Valentin, of Ciliary Motions in Reptiles and Warm-Blooded Animals. With Remarks and Additional Experiments

**Published:** 1836-04

**Authors:** 


Art. VI.—*Account of the Discovery, by Purkinje and Valentin, of Ciliary Motions in Reptiles and Warm-blooded Animals. With Remarks and Additional Experiments.* By William Sharpey, m.d. f.r.s. &c. *Edinburgh:* 1835. 8vo. pp. 16.

The experimental physiologist, in pursuing his enquiries into the wonderful phænomena of generation, is driven to the confession that Nature seldom permits man fully to comprehend her mysteries. Among other doubtful and disputed points, are the means by which the impregnated ovulum is transmitted through the Fallopian tubes. It has been assumed, but never proved, that these tubes impel forwards to the uterus the product of conception by a vermicular or a peristaltic action, similar to that of the intestines. The discovery of Purkinje and Valentin, supported as it is by the experiments of Dr. Sharpey, appears to offer a satisfactory explanation of the manner in which nature effects the transit of the ovulum to the uterus. We confine ourselves to a brief abstract of Dr. Sharpey's "Account."

A remarkable provision exists in many animals belonging to the inferior tribes, by which fluids are moved along the surface of different organs. The gills of the mussel afford a good example of this motion. If a portion of them be cut off and examined under water, the water will be perceived moving in a current along the surface in a constant and determinate direction; and when the piece of gill is inspected with the microscope, its surface will be found covered with minute hair-like organs, or cilia, in a state of continual agitation or oscillatory motion, by which they impel the fluid along the surfaces. Purkinje and Valentin of Breslaw have lately ascertained the interesting fact that this provision exists also in warm-blooded animals. They have detected the ciliary motion in the internal surface of the oviduct in birds, and the Fallopian tubes in mammalia, and of the air-passages in both. As one of these gentlemen was engaged in examining a rabbit, three days after impregnation, for the purpose of finding ova in the Fallopian tube, he saw with the microscope small portions of the mucous membrane of the tube moving briskly, and whirling round their axis. These motions were also discovered throughout the whole of the uterus, and the internal organs of generation, being, however, of different degrees of intensity at different places. They were especially brisk in the tube, less so in the cervix of the uterus, still less in the conjoined parts of that organ, most lively and rapid on its lips, and very strong in the vagina. The same vibratory motions were seen along the whole oviduct of a bird, immediately after the passage of the egg. Animals of the same kind were next examined in the unimpregnated state, and in these as well as in the amphibia the same interesting discovery was confirmed. It appears, from numerous experiments, that this vibratory motion occurs only in two systems of organs: in the sexual organs of the female, and in the organs of respiration. It is general over all parts of the internal surface of these organs, in mammalia, birds, and reptiles. In serpents, lizards, birds, and mammiferous animals, the mucous membrane of the oviduct and the respiratory passages exhibits the vibratory motion throughout its entire extent, both in the impregnated and unimpregnated state, and the smallest portion is sufficient to shew it when properly examined, so that the presence of the property in question may serve as a sure test of the system to which the parts exhibiting it may belong. If it should be ultimately established that only the respiratory and genital membrane possess this property, it would afford a new fact in support of the analogy already held on many other grounds to subsist between them.

*Method of investigation.* In parts which have long cilia, as the beginning of the oviduct in birds, the motion may be perceived by spreading out a portion of membrane on the object plate of the microscope, covering it with water, and viewing it with the requisite power of the instrument.* But to be certain of detecting the phenomena, the following plan must be pursued. The animal must be examined immediately after death. A small piece of the membrane is to be cut out, with a pair of fine curved eye-scissors, and folded on itself in such a manner that the edge of the fold shall be formed by the free surface of the membrane, its adhering surface being now inside and in contact with itself. The object is then to be placed in the *microtomic compressor*,† with a little water, and gently pressed, until the folded edge is brought clearly into view under the microscope. To render the phenomenon still more conspicuous, a fluid is to be added, holding small particles of some substance in suspension; as the black pigment of the eye diffused in water, or diluted blood. The current of these particles along the margin of the fold is so strong as to strike the most unpractised eye. It is in all cases absolutely necessary

* It is requisite to command a clear magnifying power of from three to four hundred diameters.

† An instrument contrived or improved by Professor Purkinje, by which two parallel glass plates can be approximated, so as to compress a soft object under the microscope to any required degree.

that the mucous membrane alone should be employed. No portion of muscular coat or bronchial cartilage must be left adhering to it, or the experiment fails.

*Nature and character of the vibratory motions.* They are extremely rapid. Wherever they occur, they observe, like the currents which they excite, a determinate direction.* It seems highly probable that the vibratory motions are always produced by cilia. In the female genital, and in the respiratory organs of mammalia, birds, and reptiles, the cilia cannot be mistaken. When the motion is swift the cilia can only be seen by a practised eye, but when it relaxes, they can be perceived rising and falling like oars, till at last, when all movement has ceased, they stand out like stakes from the edge of the folded membrane. Their figure can then be distinctly seen, tapering from the base to their excessively fine and delicate point: their substance is clear, with no appearance of granular structure: their consistence is very soft and tender, so that they are easily destroyed. Animal heat exerts no special influence over the vibratory motions; they are equally brisk in parts that have long been cold, as in those which are quite warm. In the three higher classes of vertebrata, the motion is not only of sufficient force to propel small particles immediately adjacent to the surface, but small portions of the mucous membrane, when detached, move themselves through the fluid. In the mussel the motion is more durable than in the vertebrata. In them it continues to exist with undiminished vigour when they are semi-putrid, softened, and macerated. Although the vibratory motion is to be regarded more as a general morphological phenomenon, yet its particular uses are not to be overlooked. By its means the secretions of those mucous membranes on which it occurs may be conveyed onwards, and many singular phenomena may perhaps be thus accounted for.†

Dr. Sharpey has repeated the experiments of Purkinje and Valentin, and confirmed their discovery of the ciliary motion in mammalia, birds, and perfect reptiles. His additional observations have also shewn, in certain cases, the direction in which matters are impelled along the surface, a point not referred to by the foreign physiologists in their preliminary memoir. Dr. Sharpey has not yet published the whole of his experiments on the subject, as he intends to review it at greater length in an article which he is preparing for the Cyclopædia of Anatomy and Physiology.

* An exception is noticed to this rule in the gills of the river mussel.

† From Müller's Archiv. Heft. i. 1835, p. 159. "In our work De Phænomeno, &c. p. 77, we have shown that the most powerful narcotics, as hydrocyanic acid, strychnine, morphium, &c. locally applied, produce no perceptible effect on the ciliary motions. And although we had also proved that these motions are altogether independent of the integrity of a larger or smaller portion of the nervous system, or even of the whole of this, it was still quite conceivable, that narcotic poisons, which, locally applied, produce little or no effect, but which, when taken into the course of the circulation, do so in the most striking manner, might, in this way, put a stop to the ciliary motions. Our recent experiments, however, contradict this in the most decided manner. We destroyed rabbits and pigeons by means of hydrocyanic acid and strychnine, sometimes by introducing it into the stomach, sometimes by applying it to recent wounds in the skin. In no case did the ciliary motions exhibit the least alteration; we took the precaution not to open any of the animals until after all convulsive movements had ceased to be perceptible in any part of the body, and even not until the limbs, when stimulated, ceased to exhibit any automatic movements. And still further to avoid all risk of error in the experiments with the pigeons, we killed a second animal of the same age, by bleeding to death. The differences which presented themselves, in all these experiments, were merely such as originated in the peculiarities of the individual animals from age, nature of the parts, &c. The want of any result from intoxication was everywhere the same." —*Rev.*

